# Comorbidity Assessment Is Essential During COVID-19 Treatment

**DOI:** 10.3389/fphys.2020.00984

**Published:** 2020-08-04

**Authors:** Shweta Jakhmola, Omkar Indari, Budhadev Baral, Dharmendra Kashyap, Nidhi Varshney, Ayan Das, Sayantani Chatterjee, Hem Chandra Jha

**Affiliations:** Discipline of Biosciences and Biomedical Engineering, Indian Institute of Technology Indore, Indore, India

**Keywords:** COVID-19, SARS-CoV2, comorbidities, heart disease, diabetes, medications, hypertension, mortality

## Abstract

Coronavirus disease 2019 (COVID-19) caused by SARS-CoV2 is associated with various comorbidities; cardiovascular diseases, hypertension, diabetes, liver, lung diseases, and neurological ailments. The majority of the dysfunctions mentioned above are often associated with endothelial deterioration, indicating that endothelium can be the target of SARS-CoV2. Our study is an exclusive observational study that quantitatively analyses COVID-19 related comorbidities. We retrieved the data of % population of COVID-19 hospitalized and deceased patients with associated comorbidities from publicly accessible portals of the five European countries. A two tailed *t*-test enabled us to determine the significant proportions of deaths compared to hospitalized patients with associated comorbidity. Our study revealed that deaths associated with cardiovascular diseases and diabetes are highly significant (*p* < 0.0001) compared to hospitalized in countries like Italy, France, and Spain unlike the Netherlands. Deaths from kidney diseases (Italy- *p* < 0.0001; Sweden- *p* < 0.0001; Netherlands- *p* = 0.0001; France- *p* = 0.0033) and neurological ailments (France- *p* = 0.0001; Netherlands- *p* < 0.0001) are significantly higher than the total hospitalized patients affected by the particular comorbidity. We have noted that deaths due to liver diseases are least associated with COVID-19 among all comorbidities. Intriguingly, immunodeficiency shows mixed outcomes in death proportions compared to the hospital admitted individuals. Besides, the treatment regime involves drugs like losartan, ACE inhibitors, angiotensin-receptor blockers, Remdesivir, Chloroquine, Hydroxychloroquine, etc. may modulate the severity of the comorbidities. These comorbidities can create chaos in the existing healthcare system and may worsen the disease outcome.

## Introduction

The severe acute respiratory syndrome coronavirus2 (SARS-CoV2) causative agent of the coronavirus disease of 2019 (COVID-19), belongs to the *coronaviridae* family. The first outbreak of SARS-CoV2 infection is associated with the wet markets of Wuhan, China, suggesting the zoonotic origin of this virus (Mackenzie and Smith, 2020). Although SARS viruses are known to infect animals, in certain cases, the viruses may cross species barrier and infect humans (Rabi et al., 2020). Since the initial outbreak of COVID-19, it has become a serious threat to public health worldwide (Rabi et al., 2020). As of 5^th^ July, 2020 COVID-19 has spread to more than 216 countries and territories, with 11,046,917 confirmed cases and 526,465 deaths across the globe. Nonetheless, European regions report 199,510 deaths with more than 2 million confirmed cases (WHO, 2020 COVID-19 dashboard, July 5^th^, 2020).

COVID-19 symptoms majorly include breathing difficulty, lung infection, pneumonia, and fever (Aghagoli et al., 2020). Emerging data depicts that COVID-19 patients have various aspects associated with hospitalization and severity. Nevertheless, the risk factors associated with SARS-CoV2 infection can be one or more such as cardiovascular diseases, hypertension, lung diseases, diabetes, etc. which may enhance susceptibility, complications, and mortality (Clerkin et al., 2020; Guo et al., 2020; Richardson et al., 2020; Yang et al., 2020). Interestingly, an increase in the number of COVID-19 patients with comorbidities is reported (Guo et al., 2020). The reasons for the exclusive relation of certain comorbidities with COVID-19 may be many. It is reported that the virus attacks the smallest of the blood vessels’ endothelial lining, thereby leading to circulation problems and impaired vessels functioning in various organs (Sardu et al., 2020). This can also explain the high vulnerability of patients with comorbidities like hypertension, obesity, diabetes, and cardiovascular disease. Besides, renal histopathological findings of COVID-19 patients show damaged endothelium (Su et al., 2020). Additionally, studies have established the presence of inflammatory cells and viral elements within the endothelial cells of COVID-19 patients. Bilateral diffuse alveolar damage with cellular fibromyxoid exudates and pulmonary oedema is observed in post-mortem samples of COVID-19 patients (Xu et al., 2020). Therefore, the multi-organ failure observed in COVID-19 may be due to damage to the endothelium caused by either direct virus infection or immune cells recruitment to the site of infection (Zaim et al., 2020). Fascinating studies also suggest an enhanced death rate for COVID-19 patients who possess one of the comorbidities mentioned above. A summarized report addressed that the overall case-fatality rate (2.3%) increased for COVID-19 patients with already prevailing comorbid conditions such as cardiovascular disease (10.5%), diabetes (7.3%), chronic respiratory disease (6.3%) and hypertension (6.0%) (Wu and McGoogan, 2020). A prospective cohort study related the prevalence of acute kidney injury in COVID-19 patients with in-hospital mortality (Cheng et al., 2020). Moreover, considering various reports on COVID-19 comorbid hospitalized cases and deaths we quantitatively analyzed the relation of hospitalized cases and death reports with one or more comorbidities in this pandemic situation.

Furthermore, there might be a correlation between COVID-19 medications and deaths due to comorbidity in COVID-19. The medication regimen may vary among countries. Notably, it increases perplexity in this concern. Thus death reports associated with particular comorbidity may also vary among countries. Hence, there is a need for prudent selection of medication for COVID-19 patients with already prevailing comorbidities. Our study provides a glimpse of the need for repurposed COVID-19 drugs on associated comorbidity based on previous studies. This may further help for better design of the COVID-19 medication regimen in patients with prevailing health conditions.

## Materials and Methods

### Data Collection

We acquired the data of COVID-19 patients with associated comorbidities from countries, namely France, Italy, Netherlands, Spain, and Sweden. For this, the publicly accessible portals or a worldwide authorized platform of each country was considered as per information availability (Epidemiology for Public Health, 2020; European Centre for Disease Prevention and Control, 2020; ISCIII, 2020; Public Health Agency, 2020; Public Health France, 2020; RIVM, 2020). Namely, the open access portals of European Centre for Disease Prevention and Control (ECDC), French Public Health Agency, Italian National Institute of Health, National Institute of Public Health and the Environment Netherlands, The Institute of Health Carlos III Spain, The Public Health Agency of Sweden have been accessed. The information about numbers of hospitalized or deceased COVID-19 patients with associated comorbidities from individual countries was already provided in their respective reports. We selected, assembled, and categorized the data based on hospitalized and deceased COVID-19 patients with associated comorbidities in Table 1. One patient may have been included in more than one comorbidity group.

**TABLE 1:** Comorbidities associated with COVID-19 related hospitalized cases and deaths.

**Conditions**		**Countries**														
		**Italy**			**France**			**Netherlands**			**Sweden**			**Spain**		
		**Hosp**	**Death**	***P* value**	**Hosp**	**Death**	***P* value**	**Hosp**	**Death**	***P* value**	**Hosp**	**Death**	***P* value**	**Hosp**	**Death**	***P* value**
		***N* (%)**	***N* (%)**		***N* (%)**	***N* (%)**		***N* (%)**	***N* (%)**		***N* (%)**	***N* (%)**		***N* (%)**	***N* (%)**	
Heart Diseases	Cardiovascular diseases	219 (21)	1568 (77)	<0.0001	612 (19)	118 (33)	<0.0001	2635 (48)	874 (50)	0.1468	389 (34)	596 (54)	<0.0001	19565 (36)	6650 (53)	<0.0001
	Hypertension	511 (49)	1410 (69)	<0.0001	202 (35*)	11 (26*)	0.231^#^	NA	NA	–	426 (37)	NA	NA	NA	NA	–
Diabetes		177 (17)	647 (32)	<0.0001	797 (25)	112 (31)	0.0134	1258 (23)	413 (24)	0.3906^#^	279 (24)	277 (25)	0.5814^#^	10239 (19)	3458 (27)	<0.0001
Chronic lung diseases		41 (4)	460 (23)	<0.0001	515 (16)	84 (23)	0.0007	1342 (25)	419 (24)	0.4008^#^	162 (14)	191 (17)	0.0492	6439 (12)	2165 (17)	<0.0001
Kidney diseases		31 (3)	431 (21)	<0.0001	181 (6)	35 (10)	0.0033	414 (8)	185 (11)	0.0001	38 (3)	187 (17)	<0.0001	NA	NA	–
Liver diseases		31 (3)	81 (4)	0.1619^#^	6 (1*)	0 (0*)	-	65 (1)	15 (1)	1	8 (1)	13 (1)	1	NA	NA	–
Immunodeficiency		NA	5 (0.20)	–	200 (6)	32 (9)	0.0264	84 (2)	16 (1)	0.0058	61 (5)	59 (5)	1	NA	NA	–
Neurological diseases		NA	307 (15)	–	102 (3)	24 (7)	0.0001	273 (5)	216 (13)	<0.0001	12 (1)	NA	–	NA	NA	–
Total comorbidity associated hospitalized cases/deaths		1043	2041	–	3192 OR 578*	360 OR 43*	-	5462	1732	–	1141	1109	–	55039	12642	–

*The table represents country-wise COVID-19 patients with associated comorbidities in numbers (N) as well as percentage (%). All the percentage values are rounded up. One patient may belong to several other groups. For France, Italy, and Sweden the hospitalized patients’ data was available and considered for intensive care unit (ICU) patients only. While for Spain the hospitalized plus ICU patients and for Netherlands patients admitted in hospital was considered in the category of hospitalized patients. The count of patients with other than enlisted comorbidities presented in the original reports have not been included in the table but considered in the total. NA, Not available; Hosp., Hospitalized patients. (Timespan for country wise data: Italy- Hosp.-NA, death- 21/02/2020–23/04/2020; France- 16/03/2020–21/04/2020; ^∗^France comorbidities data collected from 04/04/2020 (578 cases including 43 deaths); Spain-31/01/2020–22/04/2020; Netherlands-27/02/2020–10/04/2020; Sweden- 13/04/2020–19/04/2020). Neurological disease = Neuromuscular + neurovascular diseases; Cardiovascular disease = Ischemic heart disease + Atrial fibrillation + Heart failure + Stroke (Italy); Chronic lung disease = respiratory disease (spain)/ pulmonary disease (France); Immunodeficiency = HIV (Italy), Neurological disease = Dementia (Italy). ^#^*p* > 0.05-not significant; *p* < 0.05-significant calculated from *t*-Test two-tailed.*

The retrieved data ranges within a time interval until 23^rd^ April 2020. Specifically, for France, Netherlands, Spain, and Sweden the timespan for hospitalized as well as death data was 16/03/2020 to 21/04/2020, 27/02/2020 to 10/04/2020, 31/01/2020 to 22/04/2020 and 13/04/2020 to 19/04/2020, respectively. In the case of France, the data for comorbidities like hypertension and liver diseases was available for 16/03/2020 to 04/04/2020 period only. For Italy, the deceased patient’s data was obtained from the report of the Italian National Institute of Health, the timespan for which was 21/02/2020 to 23/04/2020. The hospitalized patients data was absent in this report, so it has been obtained from ECDC rapid risk assessment report 8^th^ update on COVID-19 where timespan for patient’s data is not available. Google Translate, 2020^®1^. We have also verified this translation results using other platforms like Bing Microsoft Translator, 2020^2^ and Yandex.Translate, 2020^3^.

We performed a literature survey for adverse effects of medications generally given to individuals with included comorbidities or for COVID-19 treatments. These medications and their possible mechanism have been included in Table 2.

**TABLE 2:** Comorbidities associated with COVID-19 patients and respective drugs with possible mechanisms which may show impact on these patients.

**Condition**	**Drugs**						
	**Losartan and other ACE inhibitors (Teixeira et al., 2002; Ye et al., 2002; Grein et al., 2020; Patel and Verma, 2020)**	**ARBS (Patel and Verma, 2020)**	**Chloroquine (Teixeira et al., 2002; Malviya, 2020)**	**Hydroxy- Chloroquine (Teixeira et al., 2002; Malviya, 2020)**	**Ruxolitinib (Low et al., 2015; Kerget et al., 2017)**	**Remdesivir (Grein et al., 2020)**	**Tocilizumab (Jewell et al., 2016)**

	**Possible mechanisms for adverse outcomes**						

	**Up-regulation of ACE2, Causes renal injury, Affects peripheral and sympathetic nerve activity**	**Up-regulation of ACE2**	**Induce arrhythmias**	**Induce arrhythmias**	**Development of pulmonary complications**	**Causes renal injury**	**Affects liver function, symptomatic neurological side effects**

Cardiovascular diseases	**√**	**√**	**√**	**√**	–	–	–
Hypertension	**√**	**√**	**√**	**√**	–	–	–
Diabetes	**√**	**√**	–	–		–	–
Chronic lung diseases	–	–	–	–	**√**	–	–
Kidney diseases	**√**	–	–	–	–	**√**	
Liver diseases	–	–	–	–	–	–	**√**
Immunodeficiency	–	–	–	–	–	–	–
Neurological diseases	**√**	–	–	–	–	–	**√**

*ARBs, Angiotensin-receptor blockers.*

### Statistical Analysis

A two-sample *t*-test between the proportions of hospitalized and deceased patients with various comorbidities (heart diseases, diabetes, kidney, liver diseases, immunodeficiency, and neurological disorders) from different countries (France, Italy, Netherlands, Spain, and Sweden) was performed separately to determine whether there was a significant difference between hospitalized patients and the subsequent deaths due to particular comorbidity with respect to the percent of affected individuals in the respective country. This was done using MedCalc statistical software (Schoonjans, 2019- MedCalc’s Comparison of Proportions Calculator). The *t*-statistic was significant at the 0.05 critical alpha level, *p* < 0.05 at CI-95%.

## Results

The number of hospitalized COVID-19 patients with at least one comorbidity along with the death reports, as a consequence of the disease, from various countries is enlisted in Table 1. The death proportions due to Heart Diseases including, cardiovascular diseases and hypertension, were significantly higher (*p* < 0.0001) compared to the total hospitalized patients in Italy, Sweden, and Spain. Although the proportion of death reports with respect to hospitalized patients is higher while not significant (*p* = 0.1468) for the Netherlands. In addition, the estimated deaths due to cardiovascular diseases are significantly (*p* < 0.0001) higher than hypertension (*p* = 0.231) in France. The population of deceased diabetic patients to hospitalized patients is highly significant (*p* < 0.0001) in Italy and Spain, unlike the Netherlands (*p* = 0.3906) and Sweden (*p* = 0.5814). However, France shows significance in death reports due to diabetes (*p* = 0.0134) compared to the hospitalized population. Furthermore, lung diseases being most widely associated with COVID-19 death influence a majority of individuals in various countries. However, the deaths due to lung disorders are significantly higher compared to those hospitalized in countries like Italy (*p* < 0.0001), Spain (*p* < 0.0001), France (*p* = 0.0007), and Sweden (*p* = 0.0492). Further, the Netherlands has higher while no significant (*p* = 0.4008) deaths due to chronic lung disorder compared to hospitalized patients. Moreover, deaths due to kidney diseases (Italy- *p* < 0.0001; Sweden- *p* < 0.0001; Netherlands- *p* = 0.0001; France- *p* = 0.0033) and neurological ailments (France- *p* = 0.0001; Netherlands- *p* < 0.0001) is greater than the total hospitalized patients affected by the particular comorbidity in these countries. We obtain a significant population of immunodeficient patients dead compared to hospitalized patients in France (*p* = 0.0264). On the contrary, we have seen a significant reduction in the number of immunodeficient deceased individuals compared to the total hospital admitted population in the Netherlands (*p* = 0.0058). Also, immunodeficiency seems to have no effect with respect to mortality on the hospitalized patients of Sweden (*p* = 1). Deaths due to Liver diseases are least affected by SARS-CoV2 infection compared to other comorbidities under investigation. The deaths due to liver diseases are not statistically significant compared to the total hospitalized patients for the same comorbidity (Italy- *p* = 0.1619; Netherlands- *p* = 1; Sweden- *p* = 1).

In Table 2, the drugs which are used to treat the particular comorbidity or for COVID-19 treatment and further the possible adverse outcomes are enlisted. Mainly the drugs were Losartan and other ACE inhibitors (ACEi), angiotensin-receptor blockers (ARBS), Chloroquine, Hydroxychloroquine, Ruxolitinib, Remdesivir, and Tocilizumab. Surprisingly, most of the drugs were affecting patients with cardiovascular diseases and hypertension, followed by diabetes, kidney, and neurological diseases.

## Discussion

According to a study, approximately 25% of COVID-19 positive people had at least one comorbidity associated (Guan et al., 2020). The major COVID-19 related comorbidities, namely hypertension, cardiovascular diseases, diabetes, kidney diseases, and neurological complications in various countries, are evaluated in the present article. In addition, the majority of the dysfunctions mentioned above are often associated with endothelial deterioration, indicating that endothelium can be the target of SARS-CoV2 (Chen et al., 2020).

Our study reveals that the country-wide pattern of COVID-19 associated comorbidities in hospitalized and deceased patients remains evidently similar (Table 1). Notably we found that heart diseases, including hypertension along with cardiovascular diseases, are the most frequent association with SARS-CoV2 infection in most countries (Italy, France, Spain, and Sweden) except the Netherlands. Similar reports of a high prevalence of cardiovascular disease and hypertension among hospitalized and diseased patients were demonstrated by Richardson et al. (2020), Wu and McGoogan (2020), and Zhou et al., 2020 in case reports from China and the United States, respectively. Importantly, SARS-CoV2 associated pneumonia in heart patients may develop hypoxemia due to hindrance in gas exchange (Li B. et al., 2020). This may drive complications in cardiac functions and increase the risk of death. Interestingly, our study depicts a profound proportion of deaths due to heart diseases, including cardiovascular diseases and hypertension. Also, our investigation reflects that the majority of the drugs used in the case of COVID-19 treatment are related to heart ailments, such as cardiovascular diseases and hypertension (Table 2). Further, ACE2, serine protease 2 (TMPRSS2), basigin (CD147), cathepsin B and L and sialic acid receptor expressed by the endothelial cells are used by SARS-CoV2 to gain entry into the host (Sardu et al., 2020). Besides, ACEi/ARBs exert protective effects on patients with hypertension and heart disorders; however, the effect diminishes on the binding of virus spike protein to ACE2 (Sardu et al., 2020). Chloroquine and Hydroxychloroquine, widely used drugs for SARS-CoV2 infection, were also reported to induce arrhythmias along with atrial and ventricular antiarrhythmic effects (Teixeira et al., 2002; Malviya, 2020). Intriguingly reports from government medicine agencies of most of the countries like Italy, Spain, and France (Italian Medicines Agency, 2020; National Agency for the Safety of Medicines and Health Products, 2020; Spanish Agency for Medicines and Health Products, 2020) have earlier advised the use of Chloroquine and Hydroxychloroquine against COVID-19.

Further, the virus is known to initiate diabetes by intensifying the condition of insulin resistance or by targeting the pancreatic islets. ACE2 can serve as a therapeutic target and may aid in microcirculation in the islets (Sardu et al., 2020). Several studies have already demonstrated that diabetic patients have increased susceptibility to COVID-19 (Remuzzi and Remuzzi, 2020). Nevertheless, our study shows that the population of deceased diabetic patients to the total hospitalized patients with diabetes is higher in Italy and Spain, unlike the Netherlands and Sweden.

A case report of 5700 SARS-CoV2 infected patients from New York have reported an association of chronic lung disorders with COVID-19 (Richardson et al., 2020). Our data from included countries also depicts similar observations in COVID-19 patients. Importantly the deaths due to lung pathologies are significantly high compared to those hospitalized in Italy, Spain, France, and Sweden, contrary to the Netherlands. Studies suggest that the SARS-CoV2 infection may efficiently serve as an additional factor to develop severe lung pathology (Li H. et al., 2020). Hypoxia, pulmonary distress after SARS-CoV2 infection may hamper the anti-coagulating action of the endothelium by promoting thrombosis (Sardu et al., 2020; Tang et al., 2020). Treatment of such patients with Ruxolitinib, an antiviral, may result in pulmonary complications such as the rarely observed acute respiratory distress syndrome, and pulmonary hypertension exacerbations (Low et al., 2015; Kerget et al., 2017).

The electron microscopic analysis reveals viral particles in the endothelial lining of glomerular capillary loops (Su et al., 2020). These findings strengthen the association of COVID-19 with kidney disorders underlying endothelial function in it. A study showed that more than 20% of critically ill COVID-19 patients were diagnosed with acute kidney injury (Richardson et al., 2020). Our study clearly demonstrates a significant population of the deceased with kidney disorders compared to hospitalized patients for all the countries under consideration. Besides, COVID-19 patients with cytokine release syndrome may develop acute kidney injury as an outcome of elevated vascular permeability, intrarenal inflammation, volume depletion, and cardiomyopathy (Ronco and Reis, 2020). The treatment for COVID-19 includes Remdesivir and Losartan which have possible renal side-effects (Grein et al., 2020). Prior knowledge of an individual’s medical history can facilitate better treatment to COVID-19 patients rather than adding up to the disease misery.

Other comorbidities like immunodeficient conditions and neurological diseases are reported for some countries namely France, the Netherlands, and Sweden. Patients with immunodeficiency include individuals with compromised immune systems like AIDS or undergoing any immunosuppressive therapy. Notably, the antiviral therapies given to AIDS patients may slightly benefit in COVID-19 recovery as well (Härter et al., 2020). Further, since the immune system is suppressed, these patients are highly susceptible to opportunistic pathogens, including SARS-CoV2. Therefore, we obtained variations in death reports compared to hospitalized patients from different countries. Briefly, severe COVID-19 patients report acute cerebrovascular disease, conscious disturbance, and muscle injury. Plausibly SARS-CoV2 may invade the cerebrovascular and nervous system using ACE2 receptors, or the patients with *a priori* abnormal neurological conditions might be prone to SARS-CoV2 (Liguori et al., 2020). COVID-19 patients with neurological conditions may result in poor prognosis and might die due to unrevealed complications. A case study by Mao et al. (2020) demonstrated that neural manifestations were significantly more common in severe infections compared with non-severe infections. Hence, these conditions should be monitored vigilantly. Losartan and Tocilizumab recommended for COVID-19 are previously reported to have adverse neurological effects. Thus a proper combination of medication may be adopted.

Conclusively, COVID-19 associated comorbidities and deaths were ostensibly similar in some parts of the world. How fast we are able to identify the virus-induced disorder in a person with certain comorbidity is equally important. It will enable to render the person with a suitable treatment plan within a safer timeframe. Therefore, the reaction time to the pandemic is crucial. In addition, the outcomes of COVID-19 may be independent of the comorbidities; we have efficiently corroborated available data based on hospitalized and deceased COVID-19 patients with associated comorbidities. Although the information provided by all the countries is not similarly categorized. For example, in terms of hospitalized cases, the patients in ICU are included or excluded is not specified in the report from Netherlands. Information regarding comorbidity patients of several categories is not presented in the case of Spain. Many of the other comorbidities like smoking, obesity, cancer, etc. couldn’t be considered in the study due to lack of information in most reports. Furthermore, the information about hospitalization average length was not available in the reports from where the data is collected because the data was regarding on-going cases and not with respect to any completed trials or reports. Nonetheless, cardiovascular diseases and diabetes were the most prominent of all in Italy, France, and Spain. However, the pattern of the deceased population due to COVID-19 associated comorbidities, including cardiovascular diseases, diabetes, and chronic lung ailments for the Netherlands, is contrary to other countries. The possible higher association of these diseases with COVID-19 might be due to altered physiological conditions of an individual or plausibly due to side effects of the drugs used in the country as a prescription to treat COVID-19. This clearly shows that prior knowledge of an individual’s health is critical to avoid fatal repercussions of COVID-19 treatment.

## Data Availability Statement

All datasets presented in this study are included in the article/supplementary material.

## Author Contributions

HJ, SJ, OI, and BB were involved in the final development of the project and manuscript preparation. SJ, OI, and BB wrote the manuscript draft. SJ, OI, NV, BB, and HJ analyzed the data. BB, NV, OI, AD, SC, and DK extracted the data. All authors contributed to the article and approved the submitted version.

## Conflict of Interest

The authors declare that the research was conducted in the absence of any commercial or financial relationships that could be construed as a potential conflict of interest.

## Footnotes

1 https://translate.google.co.in/, a free web-based server, was used to translate non–English-language articles with data from France, Italy, Netherlands, Spain, and Sweden into English, assuming Google translations between European languages are frequently good.

2 https://www.bing.com/translator/

3 https://translate.yandex.com/
